# Walking behaviour and glycemic control in type 2 diabetes: seasonal and gender differences-Study design and methods

**DOI:** 10.1186/1475-2840-6-1

**Published:** 2007-01-15

**Authors:** Kaberi Dasgupta, Cathy Chan, Deborah Da Costa, Louise Pilote, Mirella De Civita, Nancy Ross, Ian Strachan, Ronald Sigal, Lawrence Joseph

**Affiliations:** 1 Department of Medicine, Division of Clinical Epidemiology, McGill University Health Centre, 687 Pine Avenue West, Montreal, Canada; 2 Departments of Physiology and Agricultural, Food & Nutritional Sciences, University of Alberta, 7-55 Medical Sciences Building, Edmonton, Canada; 3 Department of Geography, McGill University, 805 Sherbrooke Street West, Montreal, Canada; 4 Department of Natural Resource Sciences, McGill University, 21111 Lakeshore Road, Ste Anne de Bellevue, Canada; 5 Department of Medicine, University of Calgary, 3330 Hospital Drive NW, Calgary, Canada (RS)

## Abstract

**Background:**

The high glucose levels typically occurring among adults with type 2 diabetes contribute to blood vessel injury and complications such as blindness, kidney failure, heart disease, and stroke. Higher physical activity levels are associated with improved glycemic control, as measured by hemoglobin A1C. A 1% absolute increase in A1C is associated with an 18% increased risk for heart disease or stroke. Among Canadians with type 2 diabetes, we postulate that declines in walking associated with colder temperatures and inclement weather may contribute to annual post-winter increases in A1C levels.

**Methods:**

During this prospective cohort study being conducted in Montreal, Quebec, Canada, 100 men and 100 women with type 2 diabetes will undergo four assessments (once per season) over a one-year period of observation. These assessments include (1) use of a pedometer with a concealed viewing window for a two-week period to measure walking (2) a study centre visit during which venous blood is sampled for A1C, anthropometrics are assessed, and questionnaires are completed for measurement of other factors that may influence walking and/or A1C (e.g. food frequency, depressive symptomology, medications). The relationship between spring-fall A1C difference and winter-summer difference in steps/day will be examined through multivariate linear regression models adjusted for possible confounding. Interpretation of findings by researchers in conjunction with potential knowledge "users" (e.g. health professionals, patient groups) will guide knowledge translation efforts.

**Discussion:**

Although we cannot alter weather patterns to favour active lifestyles, we can design treatment strategies that take seasonal and weather-related variations into account. For example, demonstration of seasonal variation of A1C levels among Canadian men and women with T2D and greater understanding of its determinants could lead to (1) targeting physical activity levels to remain at or exceed peak values achieved during more favourable weather conditions. Strategies may include shifting to indoor activities or adapting to less favourable conditions (e.g. appropriate outdoor garments, more frequent but shorter duration periods of activity) (2) increasing dose/number of glucose-lowering medications during the winter and reducing these during the summer, in anticipation of seasonal variations (3) examining the impact of bright light therapy on activity and A1C among T2D patients with an increase in depressive symptomology when sunlight hours decline.

## Background

### Cardiovascular disease and the importance of glycemic control

Cardiovascular disease is the major cause of death in adults with type 2 diabetes (T2D) [1]. The high glucose levels of T2D contribute to blood vessel injury and complications such as blindness, kidney failure, heart disease, and stroke [2]. Although blood glucose levels may be assessed at a single point in time, hemoglobin A1C better reflects overall glycemic control and is widely used to guide diabetes treatment [3]. A1C, reported as the proportion of hemoglobin molecules that are glycosylated, provides an indication of ambient glucose levels over a two to three month period, the average life span of a red blood cell. The United Kingdom Prospective Diabetes Study (UKPDS) was a landmark clinical trial that demonstrated strict glycemic control (A1C of 7% achieved by UKPDS strict arms) to be associated with a greater than 25% reduction in diabetes-associated complications compared to then-conventional therapy (A1C of 7.9% achieved by UKPDS conventional treatment arm) [4,5]. A 1% absolute increase in A1C is associated with an 18% increased risk for heart disease or stroke [6]. The Canadian Diabetes Association (CDA) considers A1C levels of less than 7% to be acceptable but less than 6% to be optimal [7].

Seemingly small variations in A1C levels are thus of clinical importance. Studies from Japan [8,9], China [10], Sweden [11], the United Kingdom [12], and the United States [13] suggest that in regions with average winter temperatures below 0°C, there is a post-winter to summer A1C decline of between 0.13% and 0.6% (Table 1). Over time, a consistent yearly increase in A1C levels, even for a few weeks, may arguably exert a cumulative adverse impact on the risk of T2D complications. The issue of seasonal variation in A1C levels is not addressed by current clinical practice guidelines [7]. Given the temperature extremes in many Canadian cities, seasonal variation in A1C levels may be higher than reported in other settings. Physical activity is a determinant of A1C that may be particularly susceptible to seasonal variation. No previous study has assessed inter-relationships of A1C and physical activity across seasons.

**Table 1:** Existing studies addressing seasonal variation in glycemic control.

	**Design**	**Number**	**Women (%)**	**Location**	**Winter A1C (%)**	**Summer A1C (%)**	**Winter-summer decline in A1C (%)**
**LETTER**							

Carney et al, 2000 [12]	Retrospective cohort study using clinic records	2,080	Not reported	Northumberland (United Kingdom)	8.4	7.7	0.6 to 0.7 in women only
Asplund, 1997 [11]	Retrospective cohort study using clinic records	800	Not reported	Sweden	7.61	7.23	0.38
Ishii et al, 2001 [8]	Prospective cohort study with monthly A1C	39	69	Fukushima province, Japan	6.96 ± 0.9	6.42 ± 0.65	0.54
Sohimiya et al [9]	Prospective cohort study with monthly A1C	11	0	Japan	6.65 ± 0.12	6.25 ± 0.21	0.40

**ARTICLE**							

Chen et al, 2004* [10]	Prospective cohort study	110	25	Taiwan	7.53 ± 1.37	7.31 ± 1.29	0.21 ± 0.94%
Tseng et al, 2005 [13] Temp (°C):	Retrospective cohort study using database	285,705 veterans. Tests:	2.2	United States, various areas	8	7.8	
>10		117,665					0.07
4.4 to 10		204,001					0.08
0 to 4.4		159,485					0.24
-6.7 to 0		229,239					0.16
-15 to -6.7		77,885					0.13

*In contrast to other studies which assessed a winter-summer difference, this study was concerned with a late winter (February) vs. pre-winter (November) difference.

### Seasonal variation: activity in general and walking in particular

Seasonal variation in physical activity has been described in nondiabetic populations. For example, in a community-based study conducted in Michigan, the proportion of respondents reporting no leisure time physical activity was highest in the winter (32.5%) and fall (28.7%) and lowest in the spring (23.4%) and summer (17.0%) [14]. In a Massachussetts study, total MET-hours/day increased by 1.4 (SD = 8.4) among men and 1.0 (SD = 6.0) among women in July compared to January (24-hour physical activity recall) [15]. In one survey, adults who perceived weather as an impediment to walking were three to five times less likely to walk 120 minutes per week [16]. In a study conducted by Tudor-Locke and colleagues, during a one-year period of pedometer self-monitoring in South Carolina and Tennessee (25 participants, nondiabetic) [17], participants recorded a mean of 10,082 steps/day, with 900 more steps/day during the summer compared to the winter. Interestingly, steps/day was highest in June and August, but somewhat lower in July, possibly attributable to extremely warm July temperatures in the southern American states.

Pedometer-based assessments arguably capture nonstructured physical activity better than do activity questionnaires [18]. Capturing nonstructured activity such as walking is particularly important among adults with T2D, among whom 66% report no leisure time physical activity [19]. Chan and colleages recently used pedometer-based assessments to assess the impact of climate variables on steps/day among adults without T2D enrolled in a 12-week pedometer-based physical activity program in Prince Edward Island [20]. For an individual taking approximately 10,000 steps/day, weather-dependent changes in physical activity could reach 2,000 steps/day, as detailed below. There was a positive linear relationship between the number of steps/day and temperature with a 3% increase in steps/day for every 10°C temperature increase. Steps/day dropped by 5% over the first 5 mm of rain fall and 8% at 14 mm of rain. Gender and BMI moderated the impact of snowfall on walking. At a BMI of 20 kg/m^2^, men increased their steps/day by about 20% for the first 10 cm of snow, while women showed no significant change. At BMI values of 30 to 35 kg/m^2^, men had no change in steps/day while women at these BMI levels experienced a decline of 7 to 10%.

### Potential impact of depressed mood

In addition to imposing physical barriers to walking and other forms of activity, inclement weather may also adversely impact mood, possibly reducing motivation to remain active. Depressed mood has been associated with poor adherence to diabetes self-care behaviours [21-23], which may lead to an increase in A1C levels. Seasonal affective disorder (SAD) is a condition in which depression occurs with a decline in hours of sunlight [24]. Depressed symptomology not meeting full criteria for depression have also been shown to have a seasonal variation in nondiabetic populations [25]. The prevalence of SAD and/or seasonal variation in depressed mood have not been examined among adults with T2D. The overall prevalence of depressive symptoms in T2D is 31% [26].

### Sex differences

Both prevalence of depressed symptoms and physical activity levels differ between men and women. Men are generally less likely to report symptoms of depression [26] and more likely to be physically active [15,27-31]. Findings from Canada's National Population Health Survey demonstrate that the presence of children in the household is a significant deterrent to becoming active for women but not for men [32]. The most commonly reported barrier to women's participation in physical activity is lack of time due to family responsibilities [33-35]. There may therefore be important gender differences in seasonal variation of A1C determinants. Neighbourhood influences such as safety, lighting, and traffic flow may impact walking behaviour with safety arguably being a particularly important factor for women. In addition to gender, other individual and social factors (e.g. ethnic background, age, education) may be important in understanding walking behaviour [36].

Body weight is a critical determinant of A1C. However, although body weight has been demonstrated to increase during the winter months in the nondiabetic population, the magnitude of this increase is small (<1 kg) [10], and, if similar among adults with T2D, would be unlikely to account for seasonal variations in A1C levels. In fact, in a Japanese study involving 11 insulin-treated T2D patients, a 0.4% winter-summer A1C difference was detected and positively associated with a 2 to 3% change in body fat proportion, but no association with weight change was observed [9]. Nonetheless, during this study, participants will be weighed, given that seasonal variation in body weight has not previously been systematically assessed in a large observational study among adults with T2D. Carbohydrate intake will also be assessed through a food frequency questionnaire. Interestingly, SAD in nondiabetic populations has been associated with decreased physical activity and increased carbohydrate cravings [24].

### Significance

Examination of environmental factors related to physical activity was highlighted as a priority research area during the National Institute of Diabetes and Digestive and Kidney Diseases conference on behavioural science research in diabetes [37]. Weather may be an important environmental factor. Although we cannot alter weather patterns to favour active lifestyles, we can design treatment strategies that take seasonal and weather-related variations into account. For example, demonstration of seasonal variation of A1C levels among Canadian men and women with T2D and greater understanding of its determinants could lead to (1) targeting activity levels to remain at or exceed peak values achieved during more favourable weather conditions. Strategies may include shifting to indoor activities or adapting to less favourable conditions (e.g. appropriate outdoor garments, more frequent but shorter duration periods of activity) (2) increasing dose/number of glucose-lowering medications during the winter and reducing these during the summer, in anticipation of seasonal variations (3) examining the impact of bright light therapy on activity and A1C among T2D patients with an increase in depressive symptomology when sunlight hours decline. Depression in individuals with SAD has been shown to be responsive to bright light therapy [24].

### Hypotheses

**Figure 1:**
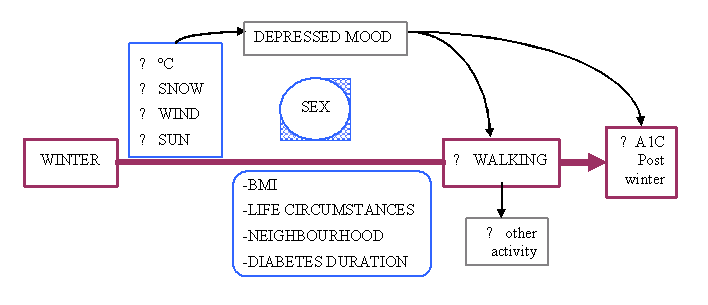
**Conceptual model**. The winter months may be associated with a decline in walking levels leading to an increase in A1C levels. Specific climate factors (low temperature, snowfall, wind chill, decreased sunlight) may account for the decline in walking. Change in walking behaviour may differ between men and women, and may be influenced by body mass index, life circumstances, neighbourhood environment, and diabetes duration. A winter increase in A1C levels may be attributable not only to a decline in walking, but also to a reduction in other activity and/or the presence of depressed mood. Seasonal variation in depressed mood has been described in nondiabetic populations. Depressed mood is associated with poor diabetes self-care and tends to occur more often among women than among men.

**Figure 2:**
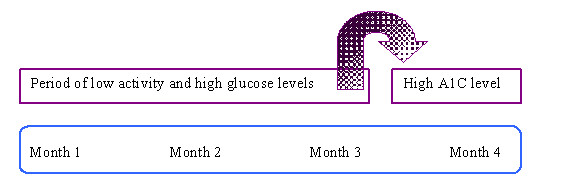
Hemoglobin A1C level reflects overall glucose control during the previous two to three month period. There is therefore a delay between low activity levels and developing a high A1C level.

As illustrated in Figure 1, factors associated with seasonal weather extremes may have an important impact on walking activity, leading to deterioration in glucose control among those with T2D. In Montreal, we postulate that the lowest levels of walking will occur during winter (January to March) because of snowfall and low temperatures. The highest levels of walking will likely occur in spring and early summer (April to June), a period of warmer temperatures and low to moderate humidity. Hemoglobin A1C (blood test) reflects overall blood glucose levels over the *previous *two to three month period (Figure 2). Therefore, highest A1C levels are expected to occur post-winter (April–May) following low levels of walking between January and March. Correspondingly, the lowest A1C levels are anticipated during the summer (July–August), following the highest levels of walking between April and June.

Lower levels of sunlight may lead to an increase in depressive symptomology which may reduce activity levels and diabetes self-care behaviours, resulting in an increase in A1C. We further expect to find women to have lower levels of walking activity than men, particularly when levels of depressed mood are high and they feel pressure to juggle competing responsibilities. Other factors that may impact walking include individual life circumstances and health status (socioeconomic status, ethnic background, BMI, diabetes duration) and neighbourhood environmental characteristics (e.g. perceived safety, availability of sidewalks). Overall activity levels may demonstrate seasonal variation and seasonal impact on A1C levels similar to that anticipated for walking behaviour.

### Objectives

Among ambulatory adults with T2D in Montreal, our primary objectives are to

1. Estimate the post winter to summer change in glucose control.

2. Determine whether this change is antedated by a winter to spring change in walking

3. Determine whether the winter to spring change in walking differs between men and women

Post winter to summer change in glucose control will be assessed primarily in terms of change in A1C level, a continuous variable. In addition, a composite dichotomous variable, increase in A1C and/or increase in glucose-lowering medication, will be examined. This is to account for the fact that in some instances, treating physicians may respond to an increase in A1C level by increasing glucose-lowering medication. As a result, A1C levels may subsequently fall. If study evaluation were to occur after medication adjustment by the treating physician, the initial deterioration in glucose control would not be detected if A1C were the only measure of glucose control employed.

Our secondary objectives are to (1) examine changes in A1C and walking between seasons (i.e. summer-fall, fall-winter, winter-spring, spring-summer) and (2) ascertain how selected variables (a) affect A1C levels and/or modify (b) walking behaviour itself and/or (c) its impact on A1C levels. These selected variables will include activity other than walking, specific climate factors (temperature, precipitation, humidity, hours of sunlight), levels of depressed mood, individual life circumstances and health status (work, care for dependents, house keeping, home maintenance, socioeconomic status, ethnic background, BMI, diabetes duration), and neighbourhood environmental characteristics (perceived safety, availability of sidewalks/walking paths, recreational facilities).

## Study Design and Methods

### Study synopsis

All participants will undergo four assessments (one per season) over a one-year follow-up period (A1C, anthropometrics, questionnaires, two weeks of pedometer monitoring), with the first assessment within three months of recruitment. The relationship between spring-fall A1C difference and winter-summer difference in steps/day will be examined through multivariate linear regression models adjusted for possible confounding. Interpretation of findings by researchers in conjunction with potential knowledge "users" (e.g. health professionals, patient groups) will guide knowledge translation efforts.

### Eligibility criteria

Participants with a history of at least one year of follow-up for T2D in Canada are eligible. Large changes in glucose control may occur during the first year after diagnosis when dietary counseling and medications are first introduced, potentially obscuring any seasonal variation. In order to permit accurate steps/day assessment with a pedometer, participants must demonstrate normal gait [38] and have a BMI ≤ 40 kg/m^2 ^[39]. Individuals with chronic conditions or medications that may compromise glycemic control are not eligible. Women who are pregnant or who are planning a pregnancy are also excluded from participation. Participants must have an identified health care provider as diabetes management will not be provided through the study. Participants must be at least 19 years of age at the time enrollment. There is no upper age limit to enrollment; however, we anticipate that more elderly individuals may be excluded on the basis of other exclusion criteria (e.g. gait abnormalities, chronic conditions/medications that may compromise glycemic control.) All questionnaires and assessments are available in both English and French.

### Recruitment

Recruitment is being conducted through McGill University-associated outpatient clinics (diabetes, internal medicine, family medicine). Participants are approached directly by our project manager, following an introduction by a member of the clinic staff (nurse, dietitian, receptionist, technician). The project manager then administers a brief questionnaire to confirm eligibility and records participant contact information.

### Assessment of glucose control

At each assessment, venous blood is sampled for A1C measurement. A1C levels may be measured in a nonfasting state. The assay being employed is the Bio-Rad Variant II high-performance liquid chromatography system (HPLC). This is the most precise A1C assay available [40]. Participants bring their current medication to each visit and the dose and type of glucose lowering agents is recorded (metformin, insulin secretagogues, thiazolidinedione, insulin).

### Walking

Steps/day is being assessed with the Yamax SW-200 pedometer, the less costly version of the Yamax Digi-Walker SW-701, which displays steps/day within ± 3% of actual steps taken [41] and provides values within ± 1% of actual steps at a treadmill speed of 80 m/min [42]. The SW-701 converts steps to calories/distance, a function not required in the present study. We have purchased 250 pedometers and have verified the accuracy of each pedometer by comparing the pedometer reading with 20 counted steps. Seventy-three percent (182) of the pedometers tested displayed a value of 20 steps; the remaining 27% (78) displayed a value that differed by no more than one step (19 or 21 steps). Participants are trained to maintain the pedometer in a vertical position, clipped to their waistband or belt. At each assessment, participants are provided with three zeroed pedometers and a padded courier self-addressed envelope. The SW-200 has a snap-on plastic cover that conceals the viewing window. Acetate Security Seals (Novovision) are placed along the margin where the cover and pedometer meet. Attempted removal of the seal results in a serrated edge, making tampering evident. This discourages participants from checking the number of steps that they register; during our observational study, we seek to alter participants' usual behaviour as little as possible with our measurement tools.

The three pedometers given to each participant at each visit are labeled as pedometer A, B, and C. During waking hours, participants (i) wear pedometer A for seven consecutive days (ii) wear pedometer B for seven consecutive days, and then (iii) mail all three pedometers to the study centre in the padded envelope (courier) provided. Pedometer C provides a measure of the additional steps recorded as a result of the mailing process. This value will be deducted from the values recorded on the other two pedometers at the stage of the analysis. We have opted for a two-week observation period per season because we believe that during a shorter period, participants may endeavour to be more active, potentially masking any seasonal differences. Participants are asked not to use the pedometer during holiday periods (Christmas, New Year's, summer vacations) as these may not be representative of usual walking patterns.

### Total activity

Given that our participants will undergo a variety of measures, it is critical that the tools used for assessment of secondary outcomes be as user-friendly as possible. Among the many questionnaires available [43], we have selected the Short Last 7 Days self-administered format of the International Physical Activity questionnaire. This two-page tool includes only seven items, but is structured to provide separate scores for walking, moderate-intensity activity, and vigorous-intensity activity. The following values are used: walking = 3.3 METS, moderate activity = 4.0 METS, and vigorous activity = 8.0 METS. The number of weekly minutes for each of these categories is derived from questionnaire responses. Test-retest reliability for this short questionnaire is good (Spearman's correlation coefficient 0.66 to 0.88) [44].

### Climate variables

Environment Canada collects and archives weather data from stations across Canada. We have opted to use data from the Trudeau station, the main weather station in Montreal. In contrast to smaller substations, at Trudeau station data are collected everyday at hourly intervals. Data are extracted from Environment Canada archives by Dr. I. Strachan (co-investigator, micrometerologist) monthly and forwarded to our project manager. Variables of interest include (maximum, minimum and average) daily temperature, relative humidity, wind speed, rainfall and snowfall. "Combination" indices such as humidex and wind chill may also be of importance. Humidex is a combination of temperature and humidity and is related to the extra difficulty in heat dissipation under hot humid air masses. Wind chill describes the extra cooling power of the wind on a body exposed to already frigid air masses.

### Depressed mood

We are using the Center for Epidemiological Studies-Depression Scale (CES-D) to assess for depressive symptoms. Compared to the Beck Depression Inventory, CES-D has fewer somatic symptoms which may overlap with physical symptoms related to chronic medical conditions rather than depressed mood [45]. Respondents indicate the frequency with which they have experienced each symptom during the past week on a 4-point scale. A score of 16 or more indicates a high level of depressive symptomology. Dr. Da Costa (co-investigator, psychologist) has used this tool previously [46,47]. The Seasonal Pattern Assessment Questionnaire (SPAQ) assesses mood states over a 1-year period and would therefore not be appropriate for seasonal tracking [25].

### Anthropometrics and dietary intake

Weight is assessed to the nearest 0.1 kg (light clothes), and height to the nearest 0.1 cm (shoes removed). Physical activity with or without weight loss may be associated with reduction in intra-abdominal adipose tissue, which may result in a decreased waist circumference [9,48]. Waist circumference is measured standing, midway between the lateral lower ribs and the iliac crests [49]. We administer the Quebec Food Frequency questionnaire at each visit. This validated tool [50] includes photographs to assist respondents in assessing quantities of food and beverage consumed.

### Other individual, social, and environmental factors

Our first visit questionnaire queries date of birth, ethnic background/country of origin, duration of residence in Canada, education, and occupation using standard questions from Statistics Canada's Canadian Community Health Survey. At all four visits, we ask about time spent in paid and unpaid labour, cooking, housekeeping, home maintenance, shopping, and childcare based on categories derived from Statistics Canada's General Social Survey reports. We enquire about medication use, other medical conditions, and diabetes duration. Three neighbourhood social and physical environment surveys have been reported in the literature to have good test-retest reliability [51] and items from these surveys, concerning local conditions favouring walking, are included in our first-visit questionnaire.

### Sample size calculation

Our main objective is to estimate the change in A1C post winter to summer. Table 2 provides the sample sizes required to accurately measure these changes, where the accuracy is given by the width of a 95% confidence interval. For example, we expect a seasonal difference of approximately 0.2% for A1C. We would like to estimate this to an accuracy of at least ± 0.15, so that the 95% confidence interval will be sufficiently distant from the null value of 0%. This will ensure that we can rule out "significant" differences that are not clinically relevant. This will require a sample size of at least 152 subjects. Our second major objective is to relate steps/day to glucose control. There are no previous studies linking steps/day with A1C levels. In our analysis, we will estimate this association via hierarchical regression models, while controlling for various possible other important factors as well as confounding factors. As a rough rule of thumb [52], for reasonably accurate estimation, between 10 and 20 subjects are required per variable added to a regression model.

**Table 2:** Sample size calculations.

**Outcome measure**	**Winter-summer difference**	**Standard deviation**	**Reference**	**Desired accuracy (95% CI width)**	**Required sample size**
***A1C***	0.207%	0.943%	See Table 1	± 0.1	342
				± 0.15	152

***Steps/day***					

Men	3,629	Estimate: 5,000	Chan et al [20] (data)	± 500	384
				± 1,000	96
				± 1,100	80
Women	1,106	Estimate: 5,000	Chan et al [20] (data)	± 500	384
				± 1,000	96
				± 1,100	80

Our third major objective is to compare the winter-summer difference in steps/day between men and women. With 80 men and 80 women we would be able to detect seasonal differences in steps/day between sexes within 1,100 steps/day (Table 2). Therefore a total sample size of 160 individuals (80 men and 80 women) would ensure estimation of a winter-summer A1C difference to within an accuracy of 0.15% while also allowing us to detect seasonal differences in steps/day between sexes within 1,100 steps/day. With 160 subjects we will be able to include between 8 and 16 independent variables in any one model. This will provide for good estimation of our primary association and reasonable control of confounding. These sample size estimates are conservative, in that they look only at two seasons even though we will have data from all four seasons. If trends over the four seasons are present, we will also be able to estimate these with reasonable accuracy.

During Dr. Chan's (co-investigator) recent study [20], participants were asked to provide 12 consecutive weeks of pedometer data. Adherence was good for the groups with which Dr. Chan conducted a baseline one-hour session, discussing the purpose of the study and the requirements of the protocol and answering questions. In these groups, 72% of participants provided complete data for 95% of the days in the 12-week period. In contrast, for the groups in which there was no face-to-face baseline information session, only 29% of participants provided complete data for 95% of the days requested. We carefully explain the demands of the protocol at baseline. Participants are asked to monitor walking for four nonconsecutive two-week periods, arguably less demanding than 12 consecutive weeks. In cohort studies led by Dr. Da Costa (co-investigator), protocol adherence was encouraged through lotteries (e.g. bookstore gift certificates, movie tickets) for those who attend follow-up visits and completed questionnaires. We conduct similar lotteries during the present cohort study. Costs of parking/transportation is also defrayed. Given these factors, we anticipate that at least 80% of participants will provide adequate pedometer data. Therefore, assuming a maximum loss to follow-up of 20%, recruitment of 200 individuals (100 men and 100 women) would result in our required sample size of 160 individuals (80 men and 80 women).

### Data Analysis

Descriptive analyses will be compiled for all variables collected in the study. Statistics will include means and standard deviations, medians and inter-quartile ranges, and proportions, as appropriate. Plots of the main outcomes (A1C, steps/day) will be created, tracking these variables over time (i.e., over seasons). Ninety-five percent confidence intervals will be calculated for changes in A1C and steps/day between seasons. Both univariate and multivariate regression models will be constructed for the association between these variables, with post winter to summer A1C difference as the dependent variable, and winter to spring/early summer difference in steps/day, as recorded by pedometer, as the independent variable. Co-variates and effect modifiers that will be considered will include gender, depressed mood, work/home responsibilities (time), socio-economic status, climate variables, BMI, and diabetes duration. In logistic regression models, the composite outcome of increase in A1C and/or increase in glucose-lowering medication will be substituted for the post winter to summer A1C difference as the dependent variable. We will investigate differences between men and women in walking activity by calculating a 95% confidence interval for the mean between-gender difference.

We will handle missing values through multiple imputation [53]. For each missing data item, we will create a regression model (typically logistic regression for missing dichotomous items and linear regression for missing continuous data) that predicts the missing item based on related non-missing items from each subject, and general trends seen in the non-missing data from all subjects. In this methodology, multiple predictions are made for each missing item, so that the uncertainty in these predictions is automatically accounted for in any analyses. Technically, multiple imputation assumes that the missing data are ignorable, which in practice assumes that the missing items are reasonably predicted by the regression models used. In addition, we will conduct sensitivity analyses to investigate the impact of having missing data that are not ignorable. Dr. Joseph (co-investigator, biostatistician) has extensive experience in developing and implementing such methods [54,55]. Because there may be considerable subject to subject variability, we will also use Bayesian hierarchical modeling. At the first level of this hierarchical model, the data from each individual over seasons will be fit. The slopes of the curves of the outcome will then be regressed on the various possible explanatory factors, including the seasons, components of the weather, BMI for each individual, and so on. This will form the second level of our hierarchical model, and the model will be completed by using low information prior distributions for all unknown parameters, allowing the information in the data to dominate the final posterior distributions [56]. We will use WinBUGS software (Version 1,4.1, Medical Research Council, Cambridge, U.K.) to estimate all posterior distributions.

### Limitations

Some participants may increase their walking as a result of being monitored. We are therefore concealing the pedometer viewing window with a snap-on cover and a special seal that make tampering evident. Requiring at least two weeks of pedometer monitoring/season should provide a pattern more representative of usual walking patterns than shorter periods. One challenge will be to ensure continued adherence to the study protocol. Careful explanation of objectives, incentives, and reimbursement of transportation costs are anticipated to maximize participation. The selected pedometers have been shown to be accurate, but must be worn correctly and require verification of position during the day. We anticipate that our pedometer training sessions and availability of study staff to respond to questions will help in this regard. Shaking the pedometer may register steps. We trust that participants will not do this deliberately, although we cannot prevent this from occurring. Given that recruitment will largely occur through a network of diabetes clinics and associations, we acknowledge that our population will most likely include individuals with regular contact with the health care system and therefore may not be entirely generalizable to those who do not receive regular care. However, even if we identified potential study candidates through random digit dialing, we believe that those with regular contact with the health system would be more likely to be aware of a diabetes diagnosis and would also be more likely to be interested in participating in a longitudinal follow-up study.

### Ethical approval

The research protocol was given approval by McGill University's Faculty of Medicine Institutional Review Board at a full board meeting on 27 February 2007 (IRB FWA 00004545). Full board approval for the addition of the Food Frequency questionnaire was provided on 28 August 2006.

### Knowledge Translation (KT)

Dr. M. De Civita, a health research psychologist with training and experience in KT theory and practice, is our KT consultant. KT encompasses a large spectrum of activities, from discovery of knowledge (*innovation*) to its implementation (*adoption*) in real-world settings. Translating our results into action will require *exchange*, *synthesis*, and *application *[57-60]. *Exchange *refers to a two-way interaction between the knowledge producers (*in-team*) and potential users of this knowledge. Our team has close links to potential users. Dr. Sigal authored the exercise recommendations in the 2003 Clinical Practice Guidelines of the Canadian Diabetes Association [61]. Dr. Dasgupta is a member of the Women Affiliate Program Advisory group, serving as their expert on women's issues in cardiovascular health. Dr. Pilote leads GENESIS, an interdisciplinary pan-Canadian research group concerned with the gender and sex determinants of cardiovascular disease of which Dr. Dasgupta is also a member. Dr. Dasgupta will hold face-to-face meetings with representatives from potential user groups, who represent the voices of patients, to discuss (a) the compatibility of research objectives and expected findings with pre-existing beliefs and attitudes and (b) the short and long-term goals for knowledge dissemination. These meetings will begin early in 2007 (7 February 2007 meeting with the West Island patient chapter of the Canadian Diabetes Association; 12 February 2007 meeting with the Women Affiliate Canadian Health Network advisory committee in Toronto, Ontario). *Synthesis and application *of in-team member's knowledge has been critical to project development and will guide the interpretation of the findings. The engagement of users in the interpretation of the findings is another aspect of the synthesis process. We will work with user group representatives to disseminate the findings to their audiences. Two meetings will be held wherein the in-team and the representatives will discuss how the knowledge should be disseminated, when such knowledge is ready for dissemination, clear communication of study limitations, and future research directions to clarify and build upon the acquired knowledge. Involvement of user groups will ensure the "contextualization" of the knowledge generated [62].

## Discussion

Extremes of temperature and other climate factors may reduce walking levels. Among individuals with T2D, this may have an adverse impact on glycemic control and, ultimately, cardiovascular risk, particularly among women. Understanding seasonal variation in walking behaviour may be a key to understanding any seasonal variation in glycemic control in T2D. Using this knowledge, in collaboration with clinicians and representatives from governmental and nongovernmental organizations, we will be better-positioned to discuss ways to develop gender-specific programs that modify perceptions of weather as a barrier to walking and/or offer season-specific activity programs. Identification of predictable seasonal changes in glycemic control could also lead to clinical practice guidelines that advocate for seasonal adjustment of glucose-lowering medication. The benefits to individuals with T2D may therefore be improved activity programs and medication recommendations, and ultimately more effective strategies for the prevention of cardiovascular disease. Just as understanding of pathophysiology is critical for the development of diagnostic tests and pharmacological agents, understanding "pathological" behaviour specific to environmental and social context is necessary for the development of context-specific, behavioural change strategies.

## Authors' contributions

The development and writing of this protocol was led by KD who secured the necessary funding. Co-authors provided expertise as follows: CC – pilot data and methodology from previous study in nondiabetic populations; DD – measurement of depressed mood; LP – approach to sex-specific analyses; MD – knowledge translation strategy; NR – measurement of social and neighbourhood factors; IS – measurement of climate variables; RS – protocol revision; LJ – statistical analyses.
